# Supplementary material for: The interactome of intact mitochondria by cross-linking mass spectrometry provides evidence for coexisting respiratory supercomplexes
Source: Mol Cell Proteomics. 2017 Dec 8;17(2):216–32. doi: 10.1074/mcp.RA117.000470 (PMC5795388; doi:10.1074/mcp.RA117.000470)
Supplement: Supplemental Data [file supp_RA117.000470_133922_0_supp_23978_fzffwf.zip › spectra_annotation/mito_DR_spectra_annotation/84-1-9-1-16-1.pdf]

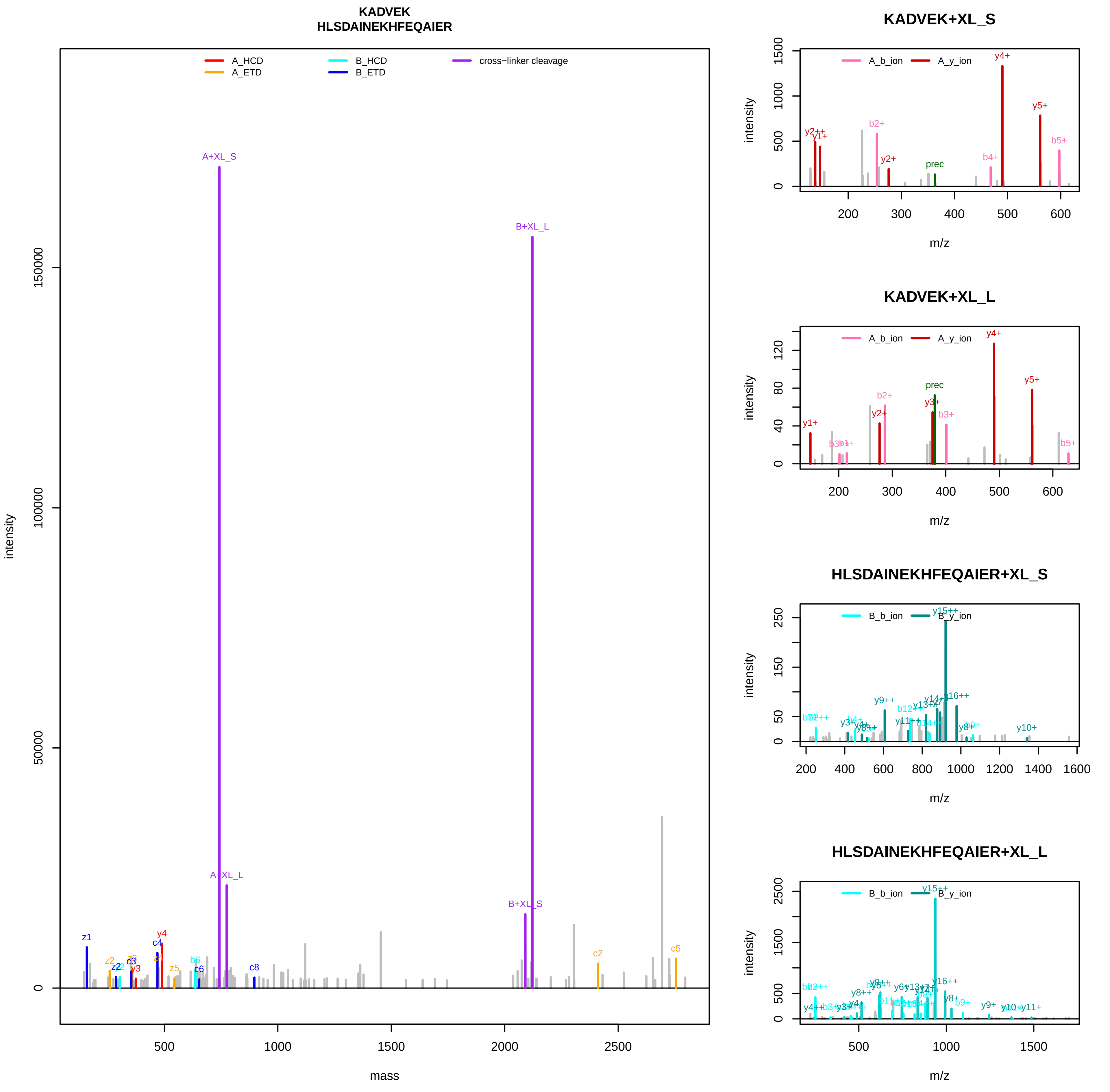
KADVEK
HLSDAINEKHFEQAIER
A_HCD
A_ETD
B_HCD
B_ETD
cross−linker cleavage
A+XL_S
B+XL_L
A+XL_L
B+XL_S
z1
z2
c3
y3
y4
c4
z5
b6
c6
c8
c2
c5
intensity
mass
0
50000
100000
150000
500
1000
1500
2000
2500
KADVEK+XL_S
A_b_ion
A_y_ion
y4+
y5+
b5+
b4+
b2+
y2+
y2++
y1+
prec
intensity
m/z
KADVEK+XL_L
y3+
b3+
y1+
HLSDAINEKHFEQAIER+XL_S
B_b_ion
B_y_ion
y15++
y16++
y9++
y10+
y8+
HLSDAINEKHFEQAIER+XL_L
y4++
y9+
y8+
b9+
